# The mitochondrial ATP synthase is a negative regulator of the mitochondrial permeability transition pore

**DOI:** 10.1073/pnas.2303713120

**Published:** 2023-12-13

**Authors:** Ryan Pekson, Felix G. Liang, Joshua L. Axelrod, Jaehoon Lee, Dongze Qin, Andre J. H. Wittig, Victor M. Paulino, Min Zheng, Pablo M. Peixoto, Richard N. Kitsis

**Affiliations:** ^a^Department of Medicine, Albert Einstein College of Medicine, Bronx, NY 10461; ^b^Wilf Family Cardiovascular Research Institute, Albert Einstein College of Medicine, Bronx, NY 10461; ^c^Department of Cell Biology, Albert Einstein College of Medicine, Bronx, NY 10461; ^d^Department of Natural Sciences, Baruch College and Program in Molecular, Cellular, and Developmental Biology, Graduate Center, City University of New York, New York, NY 10010

**Keywords:** mitochondrial permeability transition pore, mitochondrial ATP synthase, necrosis

## Abstract

The mitochondrial permeability transition pore (mPTP) is a channel in the inner mitochondrial membrane whose sustained opening in response to elevated mitochondrial matrix Ca^2+^ concentrations triggers necrotic cell death. The molecular identity of mPTP is unknown. One proposed candidate is the mitochondrial ATP synthase, whose canonical function is to generate most ATP in multicellular organisms. Here, we present mitochondrial, cellular, and in vivo evidence that, rather than serving as mPTP, the mitochondrial ATP synthase inhibits this pore. Our studies confirm previous work showing persistence of mPTP in HAP1 cell lines lacking an assembled mitochondrial ATP synthase. Unexpectedly, however, we observe that Ca^2+^-induced pore opening is markedly sensitized by loss of the mitochondrial ATP synthase. Further, mPTP opening in cells lacking the mitochondrial ATP synthase is desensitized by pharmacological inhibition and genetic depletion of the mitochondrial *cis-trans* prolyl isomerase cyclophilin D as in wild-type cells, indicating that cyclophilin D can modulate mPTP through substrates other than subunits in the assembled mitochondrial ATP synthase. Mitoplast patch clamping studies showed that mPTP channel conductance was unaffected by loss of the mitochondrial ATP synthase but still blocked by cyclophilin D inhibition. Cardiac mitochondria from mice whose heart muscle cells we engineered deficient in the mitochondrial ATP synthase also demonstrate sensitization of Ca^2+^-induced mPTP opening and desensitization by cyclophilin D inhibition. Further, these mice exhibit strikingly larger myocardial infarctions when challenged with ischemia/reperfusion in vivo. We conclude that the mitochondrial ATP synthase does not function as mPTP and instead negatively regulates this pore.

## Significance

The mitochondrial permeability transition pore (mPTP) is thought to serve homeostatic functions, but its prolonged opening in response to Ca^2+^ precipitates necrosis. Pore opening has been implicated in ischemia-reperfusion injury, cancer, neurodegenerative diseases, and aging. However, a longstanding roadblock in understanding mitochondrial-dependent necrosis and developing therapeutics has been a lack of knowledge regarding the molecular identity of mPTP. The mitochondrial adenosine triphosphate (ATP) synthase, the key enzyme responsible for ATP generation, has been hypothesized to be mPTP. We show that rather than ablating mPTP function, loss of the mitochondrial ATP synthase sensitizes pore opening and exacerbates necrotic damage in vivo. These observations indicate that the mitochondrial ATP synthase is not mPTP and provide evidence that it instead inhibits the pore.

The ability of Ca^2+^ to induce mitochondrial swelling has been known for 70 y (1) and attributed for almost 50 y to opening of a channel in the inner mitochondrial membrane (IMM) termed the mitochondrial permeability transition pore (mPTP) (2). The concept of a channel arose from biochemical (3), pharmacological (4), and electrophysiological (5, 6) studies suggesting a large, nonspecific pore whose opening is triggered by elevated mitochondrial matrix concentrations of Ca^2+^. Although not well defined, physiological functions have been ascribed to transient mPTP opening, including coupling of cardiac work with energetics (7). Most attention, however, has focused on the pathological consequences of prolonged mPTP opening, which include IMM depolarization resulting from dissipation of the electrochemical gradient and mitochondrial swelling due to the ingress of water into the solute-rich mitochondrial matrix. Mitochondrial swelling can induce outer mitochondrial membrane rupture with the release of apoptogens that promote cytosolic caspase activation and apoptosis. Alternatively, mPTP opening can trigger mitochondrial-dependent necrosis, a regulated cell death program, whose downstream events have not been delineated (8–10). Nevertheless, the relationship between mPTP opening and cell death has been convincingly demonstrated by genetic deletion (11, 12) or pharmacological inhibition (13) of cyclophilin D, a *cis-trans* prolyl isomerase in the mitochondrial matrix (14). Cyclophilin D loss/inhibition attenuates both mPTP opening and necrotic cell death through mechanisms that are not well understood (11, 12, 14).

Despite decades of investigation, the molecular identity of mPTP remains unknown. This gap in knowledge has severely hampered understanding of mitochondrial-dependent necrosis as well as the development of drugs to modulate this death program, which plays important roles in ischemia/reperfusion injury in multiple tissues, neurodegeneration, cancer, and some forms of muscular dystrophy and may contribute to aging (15). Genetic studies have excluded the necessity of voltage-dependent anion channel (16), translocator protein (also referred to as peripheral benzodiazepine receptor) (17), BCL2 associated X (BAX) and BCL2 antagonist/killer 1 (BAK) (18, 19), adenine nucleotide translocase (ANT) (20, 21), the mitochondrial phosphate carrier (22), and cyclophilin D (11, 12) for Ca^2+^-induced mPTP opening. However, it has recently been demonstrated that mPTP activation requires either ANT or cyclophilin D (21, 23).

In the last decade, several groups have suggested that the mitochondrial ATP synthase, the enzyme responsible for generation of most ATP in multicellular organisms, serves a second function as mPTP. Two models have been proposed. One involves dimers/oligomers of the 29-subunit mitochondrial ATP synthase complex (24, 25); the other posits that the c-ring of this complex forms the pore (26, 27). Genetic knockdown studies have reported that depletion of an assembled mitochondrial ATP synthase (28) or of the c-subunit (27, 29, 30) inhibits Ca^2+^-induced mPTP opening in cell lines and isolated mitochondria. Conversely, reconstitution of liposomes with biochemically purified dimers/oligomers (24, 25, 31) or monomers (32) of the mitochondrial ATP synthase or with biochemically purified c-ring (26, 30, 33) has been reported to produce channel activity with electrophysiological properties similar to those of the mPTP.

At odds with the above reports are a series of studies in HAP1 cells (nearly haploid leukemia cells) using CRISPR to ablate nuclear genes encoding various mitochondrial ATP synthase subunits. These studies show that Ca^2+^-induced mPTP opening persists in the complete absence of mitochondrial ATP synthase monomers, dimers, and oligomers achieved through targeting of individual genes encoding peripheral stalk subunits OSCP (oligomycin sensitivity-conferring protein) and subunit b (34) and supernumerary membrane subunits e, f, g, 6.8 proteolipid, and DAPIT (diabetes-associated protein in insulin-sensitive tissue) (35). Additionally, simultaneous deletion of the three nuclear paralogs encoding the c-subunit had no effect on Ca^2+^-induced mPTP opening (36). The most straightforward interpretation of these data is that an assembled mitochondrial ATP synthase or the c-ring does not function as the mPTP. An alternative interpretation that some have suggested is that there exists more than one mPTP moiety and that the lack of effects on pore function from loss of the mitochondrial ATP synthase or its c-ring is masked by redundancy provided by this second mPTP (21, 28, 29, 37).

To address this ongoing controversy, we examined mPTP function in isolated mitochondria and permeabilized cells from two HAP1 cell lines lacking an assembled mitochondrial ATP synthase and cardiac mitochondria isolated from mice that we engineered to have cardiomyocyte-specific depletion of the mitochondrial ATP synthase. In addition, to extend the analysis of mPTP function to an intact animal, we assessed myocardial infarct size during ischemia/reperfusion in vivo using the same mice. While our data confirm the previously reported persistence of mPTP in the absence of an assembled ATP synthase (34, 35), they differ in a key respect. Rather than being a neutral event, loss of the mitochondrial ATP synthase was observed to markedly sensitize isolated mitochondria and permeabilized cells to Ca^2+^-induced mPTP opening and to exacerbate tissue damage during myocardial infarction in vivo. These data demonstrate that the mitochondrial ATP synthase negatively regulates mPTP opening, a finding inconsistent with the notion that this complex functions as mPTP.

## Results

### Absence of the Mitochondrial ATP Synthase Sensitizes Ca^2+^-Induced mPTP Opening.

**Fig. 1.**
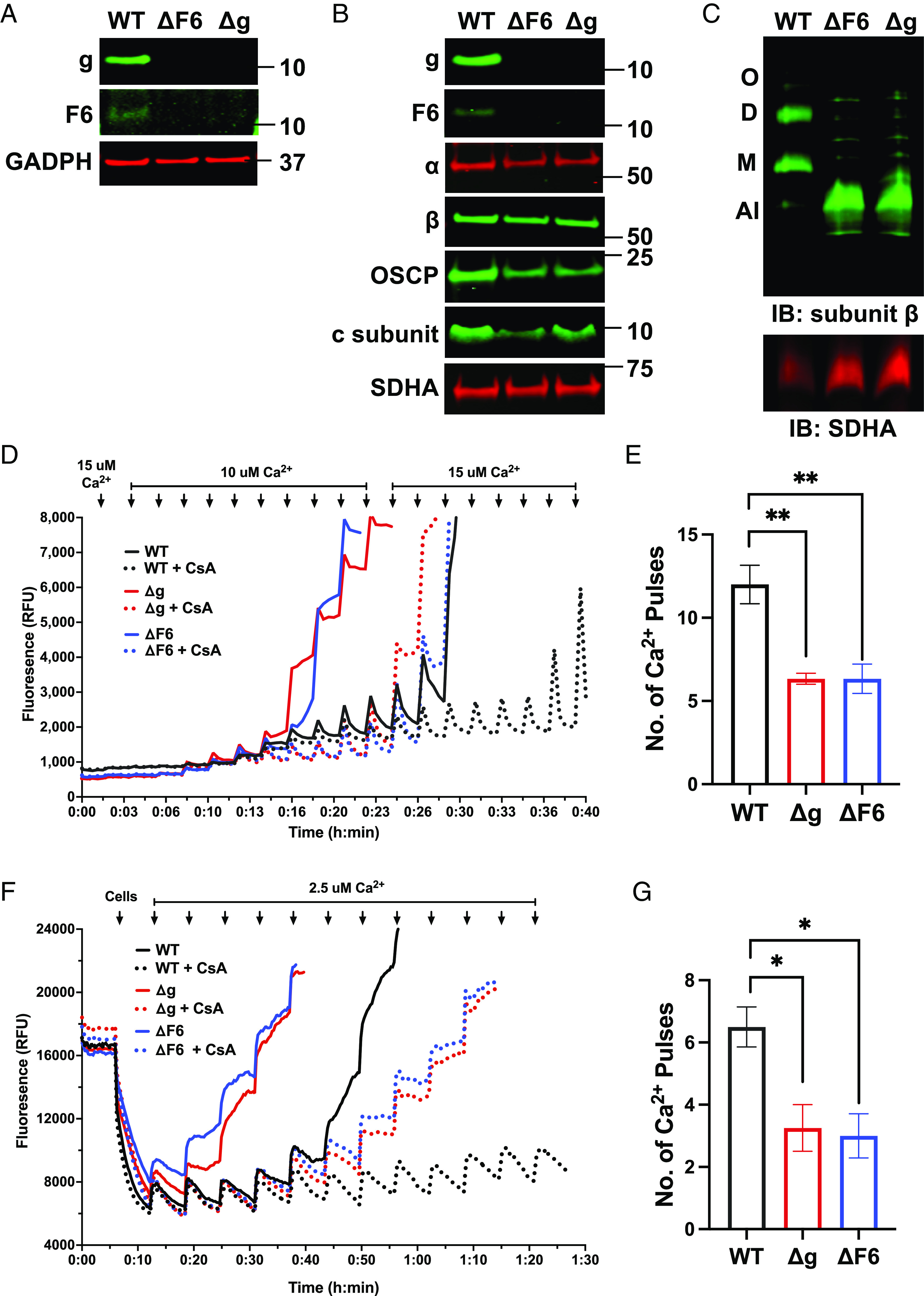
Loss of ATP synthase potentiates mPTP in HAP1 cells. Western blot of SDS-PAGE of (*A*) total cell and (*B*) mitochondrial fraction lysates from HAP1 cells. (*C*) Western blot of blue native polyacrylamide gel electrophoresis (BN-PAGE) for ATP synthase complexes extracted from mitochondrial lysates of HAP1 cells. ATP synthase complexes indicated on the left. O, oligomers; D, dimers; M, monomers; AI, assembly intermediates. Representative calcium retention capacity (CRC) assay for Ca^2+^-induced mPTP opening in (*D*) isolated mitochondria from HAP1 cells and (*F*) permeabilized HAP1 cells. Quantification of number of Ca^2+^ pulses required to induce mPTP opening in (*E*) isolated mitochondria from HAP1 cells and (*G*) permeabilized HAP1 cells. All data represent mean ± SEM, *n* = 3 to 4 independent experiments. Statistical analyses were performed using one-way ANOVA. *P < 0.05; **P < 0.01. CsA - cyclosporine A.

Previous work showed that Ca^2+^-induced mPTP opening persists in HAP1 cells deleted for the gene encoding supernumerary membrane subunit g (35), which lack an assembled mitochondrial ATP synthase (38). We began by confirming that work then extending the analysis to HAP1 cells deleted for the gene encoding peripheral stalk subunit F6, also lacking an assembled mitochondrial ATP synthase (39), in which mPTP function has not been previously studied. Immunoblotting of cell and isolated mitochondrial lysates resolved using sodium dodecyl sulfate polyacrylamide gel electrophoresis (SDS-PAGE) demonstrated the absence of subunit g or subunit F6 in HAP1-Δg and HAP1-ΔF6 cell lines, respectively, compared to wild-type HAP1 cells (HAP1-WT) (Fig. 1 *A* and *B*). Genetic knockout of subunit g resulted in decreases in the abundance of subunit F6 and vice versa as previously reported (38, 39). In addition, loss of subunit g or subunit F6 also modestly decreased levels of other mitochondrial ATP synthase subunits including α, β, OSCP, and c (Fig. 1*B*). Immunoblotting of blue native gels confirmed that HAP1-Δg and HAP1-ΔF6 cells lack mitochondrial ATP synthase monomers, dimers, and oligomers and showed the presence of an assembly intermediate that has been previously characterized as F1 in complex with the c8-ring (38) (Fig. 1*C*). Characterization of these cell lines showed that HAP1-Δg and HAP1-ΔF6 cells exhibited decreases in respiratory complexes I, III, and IV and oxygen consumption rate as previously shown (38, 39), IMM potential, and ATP levels compared to HAP1-WT cells (*SI Appendix*, Fig. S1 *A*–*D*).

We next assessed Ca^2+^-induced mPTP opening with the commonly used calcium retention capacity (CRC) assay (40). Mitochondria isolated from HAP1-WT, HAP1-Δg, and HAP1-ΔF6 cells were studied simultaneously. Successive Ca^2+^ pulses were given and pore opening scored by the sudden release of mitochondrial matrix Ca^2+^ into the buffer as detected with Calcium Green-5N, a mitochondria-impermeable dye. Despite the absence of an assembled mitochondrial ATP synthase (Fig. 1*C*), mitochondria isolated from HAP1-Δg cells underwent Ca^2+^-induced mPTP opening, as previously reported (35), as did mitochondria isolated from HAP1-ΔF6 cells (Fig. 1*D*). In contrast to previous work, however, pore opening in mitochondria from both HAP1-Δg cells (red solid line) and HAP1-ΔF6 cells (blue solid line) required approximately half the Ca^2+^ load compared to mitochondria from HAP1-WT cells (black solid line) (Fig. 1 *D* and *E*). Similar results were obtained when CRC assays were performed using digitonin-permeabilized cells rather than isolated mitochondria (Fig. 1 *F* and *G*). We conclude that mPTP function persists in both of these genetic models lacking a mitochondrial ATP synthase, but rather than having no effect, loss of the mitochondrial ATP synthase markedly sensitizes Ca^2+^-induced pore opening. Furthermore, we found that mitochondrial Ca^2+^ content was unchanged with loss of the ATP synthase (*SI Appendix*, Fig. S1*E*), indicating that changes in mitochondrial matrix calcium at baseline were unlikely responsible for the increased sensitization of mPTP.

### Cyclophilin D Knockdown Desensitizes Ca^2+^-Induced mPTP Opening Even in the Absence of an Assembled Mitochondrial ATP Synthase.

**Fig. 2.**
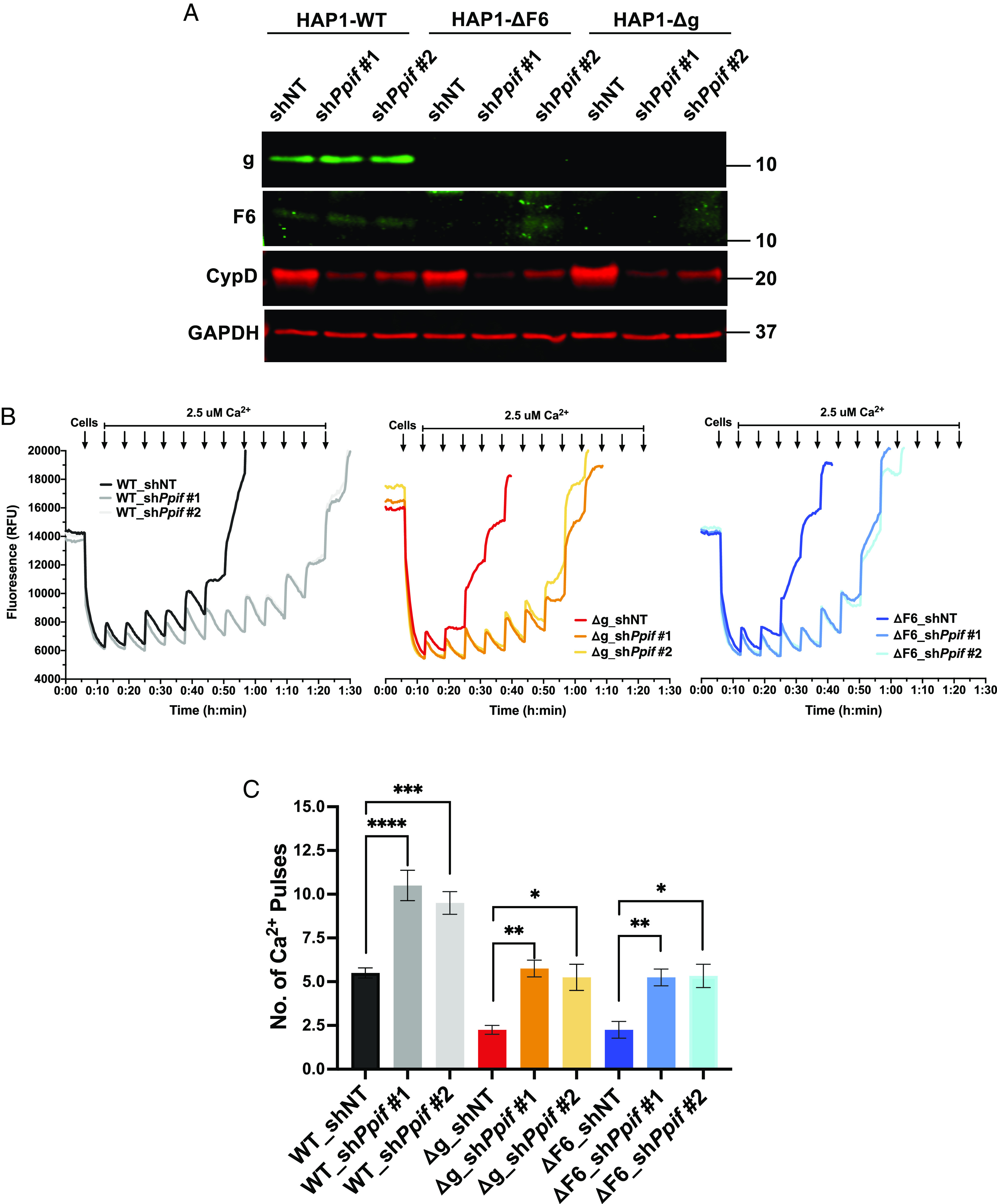
Knockdown of *Ppif* inhibits mPTP opening in the absence of ATP synthase. (*A*) Western blot of SDS-PAGE for shRNA-mediated knockdown of *Ppif* in HAP1 cells. (*B*) CRC assay for Ca^2+^-induced mPTP opening in permeabilized CypD KD HAP1 cells. (*C*) Quantification of number of Ca^2+^ pulses required to induce mPTP opening in permeabilized *Ppif* KD HAP1 cells. All data represent mean ± SEM, *n* = 3 to 4 independent experiments. Statistical analyses were performed using one-way ANOVA. *P < 0.05; **P < 0.01; ***P < 0.001; ****P < 0.0001.

The mitochondrial matrix peptidyl *cis-trans* prolyl isomerase cyclophilin D is a strong sensitizer of Ca^2+^-induced mPTP opening, although the necessity for its enzymatic activity in this function and repertoire of relevant substrates remain unresolved (11, 14, 41). While cyclophilin D binds subunit OSCP of the mitochondrial ATP synthase (24), Ca^2+^-induced pore opening in HAP1-ΔOSCP (deleted for OSCP) remains sensitive to inhibition by cyclosporine A, a small molecule that binds and antagonizes cyclophilin D and is an established inhibitor of mPTP (34). Pore opening in some other HAP1 cell lines that lack an assembled mitochondrial ATP synthase, including HAP1-Δg, has also been reported to be inhibited by cyclosporine A (35). Using both isolated mitochondria and permeabilized cell systems, we confirmed that cyclosporine A inhibits Ca^2+^-induced mPTP opening in HAP1-Δg (red dotted line) and observed the same finding in HAP1-ΔF6 (blue dotted line) (Fig. 1 *D*–*G*). However, cyclosporine A can inhibit targets in addition to cyclophilin D [e.g., calcineurin (42)]. Accordingly, we used RNAi to deplete cyclophilin D from HAP1-WT, HAP1-Δg, and HAP1-ΔF6 cells. Immunoblotting of cell lysates resolved using SDS-PAGE demonstrated knockdown of cyclophilin D with shPpif#1 and shPpif#2 (hairpins targeting transcripts encoding cyclophilin D) as compared with shNT (a non-targeting hairpin) (Fig. 2*A*). The abundance of subunit g and subunit F6 were unaffected by cyclophilin D knockdown. Using permeabilized cells, we observed that cyclophilin D depletion approximately doubled the load of Ca^2+^ needed for mPTP opening in HAP1-WT, HAP1-Δg, and HAP1-ΔF6 cells (Fig. 2 *B* and *C*). These genetic data provide strong evidence that Ca^2+^-induced mPTP opening in cells lacking the mitochondrial ATP synthase is as sensitized by cyclophilin D as in wild-type cells. This result indicates that cyclophilin D can modulate pore opening through substrates other than components in the assembled mitochondrial ATP synthase.

### Assessment of the Effects of ATP Synthase Inhibitors on Ca^2+^-Induced mPTP Opening.

Various ATP synthase inhibitors have been reported to modulate mPTP function. Oligomycin, which binds subunit c (43), has been reported to desensitize Ca^2+^-induced mPTP opening (32, 44). In contrast, benzodiazepine-243 (bz-243), which binds OSCP (45), has been reported to sensitize Ca^2+^-induced mPTP opening (24, 31). Another ATP synthase inhibitor, bedaquiline (TMC207), which binds to the interface between subunits c and ATP6 (46), has not been examined in the context of mPTP. Since our genetic cellular models indicate that loss of ATP synthase sensitizes toward mPTP opening, we wanted to determine whether these ATP synthase inhibitors would elicit the same effect on mPTP. However, in contrast to the previous studies, we observed no effect of oligomycin [at concentrations sixfold higher than those needed to suppress rates of oxygen consumption (*SI Appendix*, Fig. S1*B*)], bz-243, or TMC207 on Ca^2+^-induced pore opening in permeabilized HAP1 cells (*SI Appendix*, Fig. S2 *A* and *B*).

### Loss of the Mitochondrial ATP Synthase Does Not Affect mPTP Conductance.

**Fig. 3.**
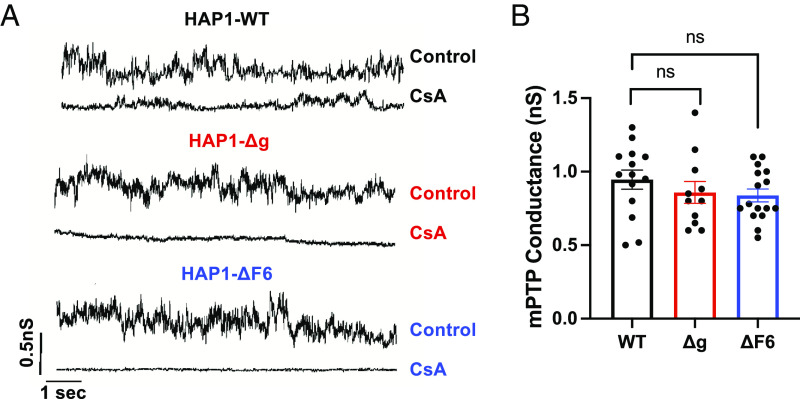
HAP1 mitoplasts depleted of ATP synthase retain mPTP conductance. (*A*) Representative patch clamp current traces of mitoplasts isolated from HAP1-WT, HAP1-Δg, and HAP1-ΔF6. The patching medium included 250 mM CaCl_2_, 5 mM succinate, and 5 mM rotenone to induce permeability transition. The high conductance openings in the WT and mutant lines were sensitive to addition of 5 to 20 µM cyclosporine A. (*B*) Quantification of mPTP conductances in HAP1 mitoplasts. Data represent mean ± SEM of 11 to 16 mitoplast recordings per group. Statistical analyses were performed using one-way ANOVA. ns, nonsignificance. CsA - cyclosporine A.

Patch clamping studies were performed in HAP1 mitochondria under Ca^2+^-stimulated energized conditions (29). The mPTP channel activities were similar in mitoplasts from HAP1-WT, HAP1-Δg, and HAP1-ΔF6 cells with average peak conductance ~1 nS, typical of mPTP (5, 6) (Fig. 3 *A* and *B*). In each case, the mPTP conductance was inhibited by cyclosporine A. Thus, in contrast to a previous study showing that deletion of subunit g in HeLa cells ablates conductance (28), the measurements here show that channel conductance is unaffected by deletion of subunit g or subunit F6 in HAP1 cells, which lack an assembled mitochondrial ATP synthase (Fig. 1*C*). These data argue against the mitochondrial ATP synthase forming the mPTP.

### Depletion of Assembled Mitochondrial ATP Synthase Sensitizes mPTP Opening in Isolated Cardiac Mitochondria and Exacerbates Myocardial Infarct Size In Vivo.

**Fig. 4.**
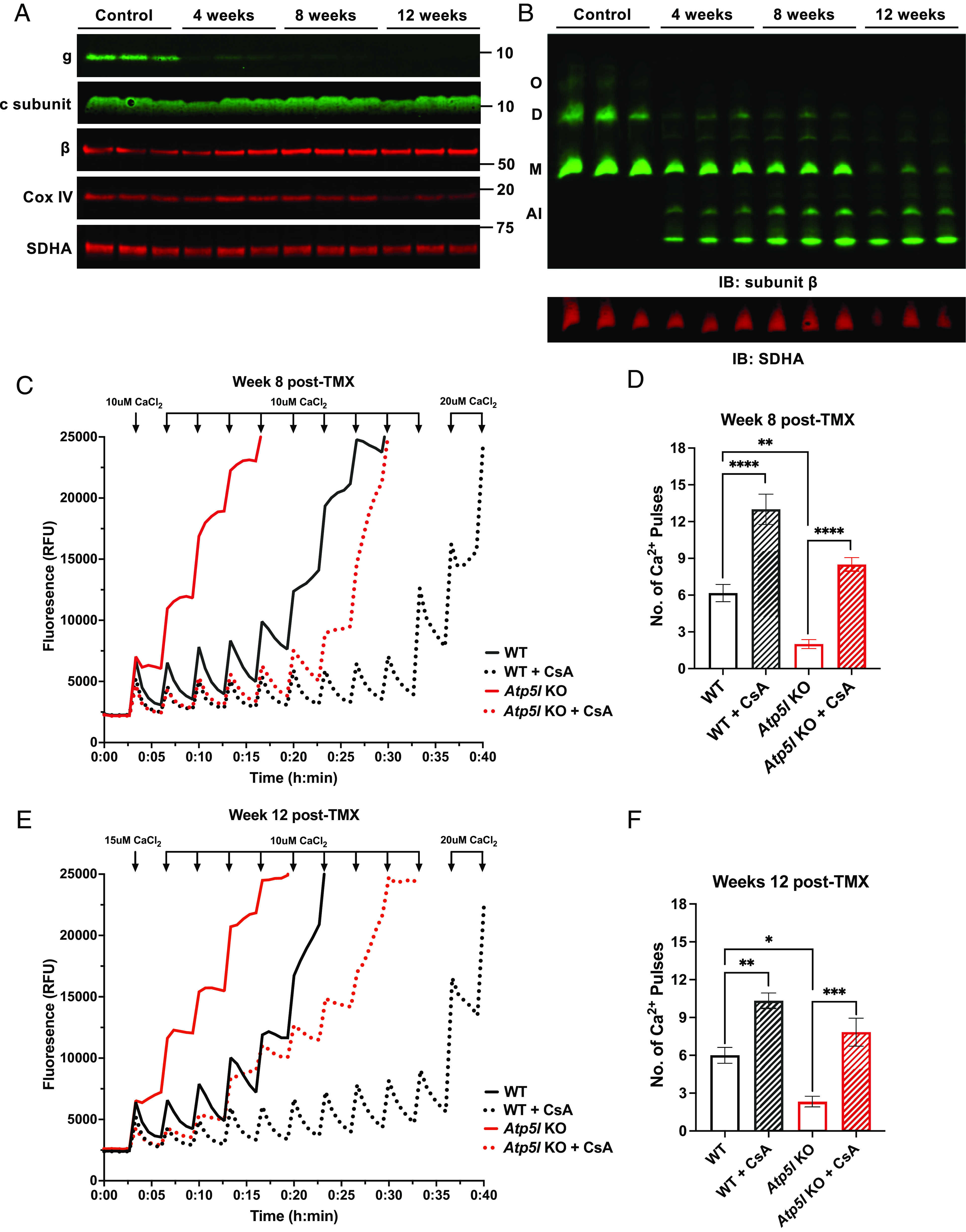
Deletion of *Atp5l* potentiates mPTP opening in cardiac mitochondria. (*A*) Western blot of SDS-PAGE for subunit g in cardiac mitochondria from WT and *Atp5l* KO mice at the indicated weeks post-TMX. (*B*) Western blot of BN-PAGE for blue native gel assessing ATP synthase complexes in cardiac mitochondria from WT and *Atp5l* KO mice indicated weeks post-TMX. ATP synthase complexes indicated on the *Left*. O, oligomers; D, dimers; M, monomers; AI, assembly intermediates. (*C*) Representative CRC assay for Ca^2+^-induced mPTP opening and (*D*) quantification of number of Ca^2+^ pulses required to induce mPTP opening in cardiac mitochondria isolated from mice at 8 wk post-TMX. (*E*) Representative CRC assay for Ca^2+^-induced mPTP opening and (*F*) quantification of number of Ca^2+^ pulses required to induce mPTP opening in cardiac mitochondria isolated from mice at 12 wk post-TMX. All data represent mean ± SEM, *n* = 6 WT mice, 6 *Atp5l* KO mice. Statistical analyses were performed using one-way ANOVA *P < 0.05; **P < 0.01; ***P < 0.001; ****P < 0.0001. CsA - cyclosporine A.

Thus far, all published studies on the role of the mitochondrial ATP synthase as mPTP have been performed using immortalized cell lines or purified proteins reconstituted in lipid membranes. We wished to extend this analysis to mitochondria from intact mice and to tissues in vivo. Accordingly, we engineered mice whose cardiomyocytes were markedly depleted of the mitochondrial ATP synthase beginning in adult life by deleting *Atp5l* encoding subunit g, which is needed for assembly of the complex (38). To accomplish this, we generated *Atp5l   *^flox/flox^; *Myh6-Mer-Cre-Mer*^tg/-^ mice and treated them with a 5-d course of tamoxifen (TMX) to initiate gene deletion specifically in cardiomyocytes at 5 to 6 wk of age. As controls, we used *Atp5l*^+/+^; *Myh6-Mer-Cre-Mer*^tg/-^ mice, also given the same TMX regimen, in which the *Atp5l* gene is not deleted. Immunoblotting of cardiac mitochondrial protein resolved using SDS-PAGE for subunit g demonstrated that this subunit was lost in knockout mice (Fig. 4*A*). We next analyzed mitochondrial ATP synthase complexes using blue native gel electrophoresis of cardiac mitochondrial protein followed by immunoblotting for subunits g, β, and c (Fig. 4*B* and *SI Appendix*, Fig. S3 *A* and *B*). These data showed that levels of oligomers and dimers were markedly reduced within 4 wk of gene deletion, consistent with the role of subunit g in dimerization of the complex (38, 47). Mitochondrial ATP synthase monomers were lost in a time-dependent manner consistent with the known ~33 d half-life of subunit g in mouse heart as measured by deuterium labeling (48) such that, at 8- and 12-wk post gene deletion, ~25% and ~10%, respectively, of wild type levels remained. In contrast to the mutant HAP1 cells (*SI Appendix*, Fig. S1*A*), levels of mitochondrial respiratory complexes I, III, and IV in mouse hearts were not altered at 8 wk post gene deletion (*SI Appendix*, Fig. S3*B*), although there was a ~30% decrease in IMM potential at this time point (*SI Appendix*, Fig. S3*C*). Mice appeared normal at rest until at least 12 wk post gene deletion. Cardiac systolic function measured at 8 wk post deletion was normal (*SI Appendix*, Table S1). The detailed molecular, mitochondrial, metabolic, and cardiac analysis of the cardiomyocyte-specific Atp5l knockout mice will be reported elsewhere.

To test whether the sensitization in Ca^2+^-induced mPTP opening that we observed in HAP1 mutants lacking an assembled mitochondrial ATP synthase is also present in cardiac mitochondria depleted of this complex, we first performed CRC assays using mitochondria isolated from cardiomyocyte-specific *Atp5l* knockout mice 8 wk post gene deletion. As in HAP1 mutants, depletion of the mitochondrial ATP synthase markedly decreased the Ca^2+^ load required for mPTP opening (Fig. 4 *C* and *D*). Additionally, Ca^2+^-induced mPTP opening in cardiac mitochondria from knockout mice remained sensitive to inhibition by cyclosporine A treatment. A similar degree of mPTP sensitization was observed at 12 wk post depletion (Fig. 4 *E* and *F*). These data demonstrate that depletion of an assembled mitochondrial ATP synthase sensitizes cardiac mitochondria to Ca^2+^-induced mPTP opening and reinforces the concept that the ATP synthase is a negative regulator of the pore.

**Fig. 5.**
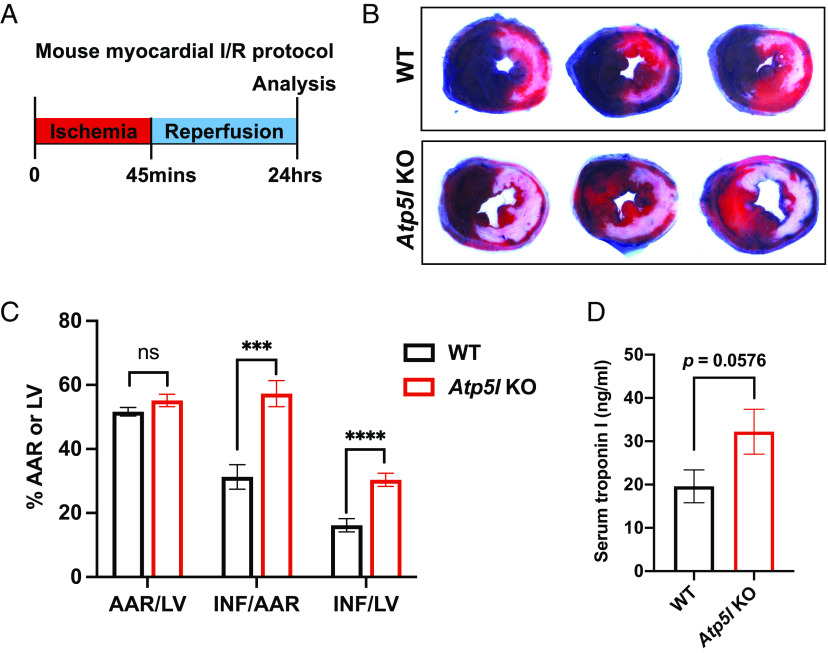
Deletion of *Atp5l* augments myocardial damage during ischemia-reperfusion injury. (*A*) Schematic for myocardial ischemia-reperfusion (I/R) protocol in WT and *Atp5l* KO mice at weeks 8 to 11 post-TMX. (*B*) Representative images of TTC staining to assess infarct size in WT and *Atp5l* KO mice subjected to myocardial I/R. (*C*) Quantification of AAR/LV, INF/AAR, and INF/LV following myocardial I/R in WT and *Atp5l* KO mice. (*D*) Troponin I release following myocardial I/R in WT and *Atp5l* KO mice. All data represent mean ± SEM, *n* = 13 male WT mice, 12 male *Atp5l* KO mice. Statistical analyses were performed using two-tailed Student’s *t* test. ***P < 0.001; ****P < 0.0001.

Prolonged opening of the mPTP triggers necrotic cell death and infarction in response to ischemia/reperfusion in multiple tissues in vivo as evidenced by attenuation of cell death and tissue damage by interventions such as cyclosporine A treatment or deletion of the gene encoding cyclophilin D (11–13). Since our data show that depletion of the mitochondrial ATP synthase sensitizes mPTP opening in cardiac mitochondria (Fig. 4 *C*–*F*), we next tested whether cardiomyocyte necrosis and myocardial infarction size would be affected. Accordingly, we subjected control and cardiomyocyte-specific *Atp5l* knockout mice to myocardial ischemia followed by reperfusion to model reperfused myocardial infarction (Fig. 5*A*). We performed these infract studies at 8 to 11 wk post gene deletion when cardiac function is normal (*SI Appendix*, Table S1). Regional ischemia was induced by surgical ligation of the left coronary artery for 45 min and reperfusion effected by releasing the suture. Ischemic area at risk and infarct size were assessed after 24 h reperfusion using Evans blue and tetrazolium staining, respectively. In addition, serum concentrations of cardiac troponin I, a cardiomyocyte-specific protein, were used as a specific marker of cardiomyocyte necrosis as employed clinically. Despite similar ischemic areas at risk, male and female knockout mice exhibited markedly larger myocardial infarctions (Fig. 5 *B* and *C* and *SI Appendix*, Fig. S4 *A* and *B*) and increased serum troponin I levels (Fig. 5*D*) compared with control mice. Thus, increases in cardiomyocyte necrosis and infarct size mirror the baseline sensitization of Ca^2+^-induced mPTP opening observed in isolated cardiac mitochondria from these mice.

## Discussion

The molecular identity of the mPTP remains unresolved. The two major questions at the forefront are a) whether the assembled mitochondrial ATP synthase or its c-ring functions as the pore and b) whether there exists more than a single mPTP-forming moiety. The focus of this study is whether an assembled mitochondrial ATP synthase serves a second function as mPTP. Genetic loss of function approaches in cells have yielded diametrically opposite answers to this question with some studies showing loss (28), and others persistence (34, 35), of mPTP function in the absence of an assembled mitochondrial ATP synthase. The reasons for these discrepant results are not clear. Further, the issue of redundancy (21, 28, 29), which limits the interpretation of loss-of-function approaches, presents a challenge for those studies showing pore persistence in the absence of an assembled mitochondrial ATP synthase to conclude with certainty that the mitochondrial ATP synthase cannot function as mPTP.

It is with respect to this last point that the current study contributes critically to this debate. Rather than loss of the mitochondrial ATP synthase being merely a “neutral” event that does not affect pore opening, our data demonstrate that Ca^2+^-induced mPTP opening is markedly sensitized by the absence of an assembled mitochondrial ATP synthase. This was observed in mitochondria isolated from HAP1 cells, permeabilized HAP1, and in isolated cardiac mitochondria. Each of these models either lacks (HAP1 lines) or is markedly depleted for (cardiac mitochondria) an assembled mitochondrial ATP synthase. In all cases, not only does Ca^2+^-induced mPTP opening persist with loss of the mitochondrial ATP synthase, it is markedly sensitized requiring ~50% of the Ca^2+^ load needed for pore opening in controls with an assembled mitochondrial ATP synthase. The fact that loss of the mitochondrial ATP synthase sensitizes Ca^2+^-induced pore opening indicates that the mitochondrial ATP synthase negatively regulates mPTP, an observation at odds with the mitochondrial ATP synthase being the pore. While this result does not exclude the concept of redundant mPTP-forming moieties, it makes it highly unlikely that the mitochondrial ATP synthase is one of these pore-forming entities. In summary, we conclude that an assembled mitochondrial ATP synthase—monomers, dimers, or multimers—does not function as mPTP and demonstrate that it is a negative regulator of this pore.

A second important advance in this study is the extension of the concept of mPTP sensitization to an in vivo setting—myocardial infarction induced by ischemia/reperfusion injury in intact mice. Cardiomyocyte mPTP opening has been linked with necrotic tissue damage (infarction) during reperfused myocardial infarction in multiple studies showing that pharmacological inhibition (13) or genetic ablation (11, 12) of cyclophilin D, an established potentiator of mPTP opening, limits infarct size. Accordingly, we deleted *Atp5l*, encoding subunit g, specifically in cardiomyocytes starting in adult life. Rather than suffering smaller infarct as would be predicted if an assembled mitochondrial ATP synthase formed mPTP, knockout mice exhibited increased cardiomyocyte necrosis and markedly larger infarcts than controls. Given the central role of mPTP opening in cardiomyocyte necrosis during reperfused myocardial infarction (11–13), these data provide the first in vivo evidence suggesting that depletion of the mitochondrial ATP synthase sensitizes mPTP opening during myocardial ischemia/reperfusion in vivo and that the assembled mitochondrial ATP synthase does not function as mPTP but instead negatively regulates this pore.

The mechanisms by which cyclophilin D potentiates Ca^2+^-induced mPTP opening are incompletely understood including which of its interactions with proteins in mitochondria are relevant to this effect (14). While prior work showing that cyclosporine A inhibits pore opening in cells lacking an assembled mitochondrial ATP synthase (34, 35) suggests that other interactors or substrates may be involved, we wished to confirm this genetically because cyclosporine A also inhibits other targets such as calcineurin (42) that may impact mPTP function (18, 49). Thus, after confirming the effects of cyclosporine A on inhibiting mPTP in isolated mitochondria and permeabilized HAP1-Δg and HAP1- ΔF6 cells, we used RNAi to stably knockdown cyclophilin D in HAP1-WT, HAP1-Δg, and HAP1-ΔF6 cells. We observed that depletion of cyclophilin D approximately doubles the Ca^2+^ load needed for mPTP opening in all three cell contexts. These pharmacological and genetic data provide strong evidence that cyclophilin D can sensitize Ca^2+^-induced mPTP opening through targets other than subunits in the assembled mitochondrial ATP synthase.

This study focuses on the role of an assembled mitochondrial ATP synthase in forming the mPTP. The mutations we employed, inactivation of the genes encoding subunits g and F6, do not allow us to address whether the c-ring can form mPTP because assembly intermediates containing the c-ring in complex with F1 persist in these models [(38, 39) and Figs. 1*C* and 4*B* and *SI Appendix*, Fig. S3*A*). Given current knowledge regarding the sequence of events in mitochondrial ATP synthase assembly (35, 38, 39), these complexes are likely integrated into the IMM. Although oligomycin has previously been reported to inhibit mPTP by binding to the c subunit (32, 43, 44), we did not observe any effect of oligomycin on mPTP opening (*SI Appendix*, Fig. S2*A*). Accordingly, we cannot rule out the possibility that the absence of an assembled ATP synthase may enhance the formation of the mPTP by c-ring complexes in our cellular and mouse models. The c-ring has been suggested to be sufficient to form the mPTP by some studies (26, 27, 29, 30, 32, 33, 44), but other work has shown that mPTP opening persists in cells deleted for all 3 paralogs—*Atp5g1*, *Atp5g2*, *Atp5g3*—encoding the c-subunit (36). Another possibility is that the F1–c8 complex sensitizes the opening of an mPTP formed by another pore-forming unit. Further studies will be needed to investigate the role of these complexes in mPTP regulation.

Although we do not know how the absence/depletion of an assembled mitochondrial ATP synthase sensitizes Ca^2+^-induced mPTP opening, we propose 3 potential mechanisms: a) The assembled mitochondrial ATP synthase interacts with and allosterically inhibits the pore-forming entity. In the case of ANT, a candidate for the pore-forming unit, an interaction is already known (50). b) A metabolite or ion whose abundance is impacted by loss of the assembled mitochondrial ATP synthase regulates mPTP sensitivity. c) Changes in mitochondrial cristae structure resulting from loss of the mitochondrial ATP synthase (51–53) may sensitize mPTP opening. This possibility is supported by studies showing that cristae abnormalities resulting from loss of OPA1 are associated with increased mPTP sensitivity (54) and exacerbation of ischemia/reperfusion-induced damage (55) while OPA1 overexpression attenuates ischemia/reperfusion injury (56).

In conclusion, the mitochondrial, cell-based, and in vivo studies presented herein reveal an unexpected role for the mitochondrial ATP synthase as a negative regulator of mPTP indicating that an assembled mitochondrial ATP synthase does not function as mPTP.

## Materials and Methods

### Cell Culture.

HAP1-WT, HAP1-Δg, and HAP1-ΔF6 cells were obtained from Horizon Discovery. HAP1 cells were cultured in Iscove’s Modified Dulbecco Media (Sigma) supplemented with 10% (v/v) fetal bovine serum (FBS) and 100 units/mL penicillin/100 ug/mL streptomycin. HEK293T cells were cultured in Dulbecco Modified Eagle’s Medium (DMEM) (Sigma) supplemented with 10% (v/v) FBS, 2 mM L-glutamine, and 100 units/mL penicillin/100 ug/mL streptomycin. All cells were cultured in a 37 °C incubator in the presence of 5% CO_2_.

### Lentivirus Production and Transduction.

To produce lentivirus, HEK293T cells were transfected with pMD2.G (Addgene 12259), psPAX2 (Addgene 12260), and pLKO.1 encoding either nontargeting shRNA (Addgene 1864) or *ppif* shRNA (Sigma TRCN0000049343 or Sigma TRCN0000049347) using XtremeGene HD (ThermoFisher) according to the manufacturer’s protocol. The supernatant was collected and concentrated using Lenti-X Concentrator (Takara Bio Inc) and stored at −80 °C until ready to use. HAP1 cells were infected with the lentivirus containing 0.8 µg/mL polybrene. Experiments were performed in HAP1 cells 3 to 7 d post infection.

### Isolation of Mitochondria for CRC Assays.

#### Mouse heart.

Under deep isoflurane anesthesia, the heart was excised and washed in ice-cold phosphate buffered saline (PBS) with 10 µM ethylenediaminetetraacetic acid (EDTA). Excess blood and buffer were removed with a paper towel, and the atria and great blood vessels were dissected away. The heart was placed in 1 mL ice-cold PBS-EDTA buffer and was finely minced with a sharp blade. The minced tissue was transferred into a 15-mL tube and 4 mL of the PBS-EDTA buffer was added. Trypsin was added and homogeneously dissolved by pipetting up and down the suspension on ice. This was repeated in 3-min intervals for a total of 15 min. Trypsin inhibitor was added at the end of the time point. Tissues were allowed to settle, and the supernatant was removed. The remaining tissue was suspended in 4 mL of ice-cold IBm1 buffer (67 mM sucrose, 50 mM tris-HCl, 50 mM KCl, 10 mM EDTA, 0.2% BSA) and was transferred in a cold 5-mL dounce homogenizer. Using a power drill, the tissue was homogenized gently. The homogenate was transferred to a 15-mL tube and spun at 800 × g for 10 min at 4 °C. The supernatants were collected into two 2-mL centrifuge tubes and spun at 10,000 × g for 10 min at 4 °C. The supernatant was removed, and the mitochondrial pellet was resuspended in 1 mL IBm2 buffer (250 mM sucrose, 10 mM tris-HCl, 10 mM KCl, 3 mM ethylene glycol-bis(β-aminoethyl ether)-N,N,N′,N′-tetraacetic acid (EGTA)-tris). The suspension was spun at 10,000 × g for 5 min at 4 °C. The mitochondria pellet was resuspended in EBm buffer (250 mM sucrose, 10 mM tris-HCl, 5 mM MgCl_2_, 0.02 mM EGTA-Tris, 2 mM KH_2_PO_4_) and concentration was determined using Bradford reagent (BioRad).

#### HAP1 cells.

HAP1 cells were cultured as described in the previous section. Cells were collected using trypsin and resuspended in PBS with 10 µM EDTA and incubated on ice for 10 min. The suspension was spun at 800 × g for 10 min at 4 °C. Supernatant was discarded and the cell pellet resuspended in ice-cold IBc1 buffer (200 mM sucrose, 10 mM Tris-MOPS, 1 mM EGTA-Tris) and transferred in a cold 5-mL dounce homogenizer. Using a power drill, the cell suspension was homogenized gently. The homogenate was transferred to a 15-mL tube and spun at 800 × g for 10 min at 4 °C. The supernatants were collected into two 2-mL centrifuge tubes and spun at 10,000 × g for 10 min at 4 °C. The supernatant was removed, and the mitochondrial pellet was washed with 1 mL Ebc buffer (10 mM Tris-MOPS, 125 mM KCl, 10 mM EGTA-Tris, 1 mM KH_2_PO_4_) and spun at 10,000 × g for 5 min at 4 °C. The mitochondria pellet was resuspended in Ebc buffer and the concentration was determined using Bradford reagent (BioRad).

### CRC Assays Using Isolated Mitochondria.

The total amount of mitochondria needed for the experiment was spun at 10,000 × g for 5 min at 4 °C. The mitochondria pellet was resuspended in Ca^2+^ depletion buffer (125 mM KCl, 20 mM 2-[4-(2-hydroxyethyl)piperazin-1-yl]ethanesulfonic acid (HEPES), 1 mM EGTA-Tris, 2 mM KH_2_PO_4_, 5 mM MgCl_2_, 15 mM NaCl, 1 mM K_2_EDTA, 0.1 mM Malate, pH 7.1) for 5 min on ice, then spun at 10,000 × g for 5 min at 4 °C. The resulting pellet was resuspended in assay buffer (120 mM KCl, 10 mM Tris-HCl, 5 mM MOPS, 5 mM KH_2_PO_4_, 0.01 mM EGTA-Tris, 10 mM Glutamate, 5 mM Malate, 1 µM Calcium Green-5N, pH 7.4) (ThermoFisher) and pipetted into black clear bottom 96-well plate. Then, 2 µM cyclosporine A (Sigma) was added and incubated protected from light for 20 min. Baseline fluorescence (485 nm/535 nm, Ex/Em) was measured kinetically. At the end of each interval, CaCl_2_ solution (concentration as indicated in each experiment) was added with a multichannel pipette to each well simultaneously and the kinetic readouts were resumed immediately using Infinite M1000 (TECAN) multiwell plate reader. This process was repeated until all groups showed a huge spike in relative fluorescence units (RFU) manifesting the loss of CRC.

### CRC Assays Using Permeabilized HAP1 Cells.

HAP1 cells were cultured as described in the previous section. Cells were collected using trypsin resuspended in PBS and counted using Cellometer Mini (Nexcelom Bioscience). Appropriate amounts of cells per group were aliquoted and treated with cyclosporine A (Sigma), oligomycin (Sigma), TMC207 (Cayman Chemicals), or benzodiazepine-423 (Sigma) for 20 min. The assay buffer supplemented with 0.002% digitonin, 0.5 µM thapsigargin, and 1 µM Calcium Green-5N pH 7.4 was pipetted into the wells and baseline was fluorescence measured (506 nm/532 nm, Ex/Em) kinetically. After the baseline readout, an appropriate volume of cells (5 × 10^5^ cells/well) were pipetted into each well and the kinetic readout was resumed immediately. CaCl_2_ solution (2.5 µM) was added with a multichannel pipette to each well simultaneously at the end of each interval and the kinetic readouts were resumed immediately using Infinite M1000 (TECAN) multiwell plate reader. This process was repeated until all groups showed a huge spike in RFU manifesting the loss of CRC.

### Evaluation of Mitochondrial Ca^2+^ Content Using Permeabilized HAP1 Cells.

Mitochondrial Ca^2+^ was evaluated using a protocol as previously described (57, 58). Briefly, permeabilized HAP1 cells were treated with MCU inhibitor Ru360 and NCLX inhibitor CGP-37157 to block mitochondrial Ca^2+^ flux. Fura2 (Invitrogen) was added to measure the extramitochondrial Ca^2+^ content. FCCP was added to uncouple and dissipate mitochondrial membrane potential to release matrix Ca^2+^. The change in fluorescence signals for Fura2 after FCCP addition reflects the mitochondrial Ca^2+^ content.

### Generation of Cardiomyocyte-Specific *Atp5l* Knockout Mice.

*Atp5l*^flox/flox^ mice on a C57BL/6N background were obtained from MRC Harwich and crossed with *Myh6-Mer-Cre-Mer*^tg/-^ transgenic mice on a C57BL/6J background to generate mice *Atp5l*^flox/flox^; *Myh6-Mer-Cre-Mer*^tg/-^ (hemizygous) mice. Controls were *Atp5l*^+/+^; *Myh6-Mer-Cre-Mer*^tg/-^ (hemizygous) mice. Mice 5 to 6 wk old were treated with TMX 20 mg/kg per dose for 5 d. Confirmation of *Atp5l* deletion and ATP synthase loss was determined at 4, 8, and 12 wk post-TMX by SDS-PAGE and BN-PAGE western blotting, respectively.

### Myocardial Ischemia-Reperfusion Injury In Vivo.

Ischemia/reperfusion was induced in mice 8 to 11 wk post-TMX as indicated for each figure by ligating the left coronary artery for 45 min followed by reperfusion for 24 h (59). Area at risk (AAR) and area of infarct (INF) were assessed by Evan’s blue dye and 2,3,5-triphenyltetrazolium chloride (TTC) staining, respectively (59). Serum concentrations of cardiac troponin I were measured using the mouse cardiac troponin I ELISA kit (Life Diagnostic Inc) according to the manufacturer’s instructions.

### SDS-PAGE Immunoblotting.

#### HAP1.

HAP1 cells were lysed by RIPA buffer supplemented with protease inhibitors (Sigma). Then, 30 µg protein was used for SDS-PAGE and western blotting. After blocking, PVDF membranes were incubated with primary antibody overnight at 4 °C and secondary antibody at room temperature for 1 h.

#### Cardiac mitochondria.

Hearts were isolated and suspended in sucrose buffer (320 mM sucrose, 3 mM MgCl_2_, 5 mM EGTA, 5 mM DTT, 1 mM phenylmethanesulfonyl fluoride (PMSF), 10 mM HEPES-KOH, pH 7.5) supplemented with protease inhibitors and homogenized with power drill (10×). Cell debris was removed after centrifugation at 600 × g for 10 min at 4 °C. Mitochondria was isolated by centrifugation at 8,000 × g for 10 min at 4 °C. Then. 30 µg of mitochondria protein was used for SDS-PAGE and western blotting. After blocking, PVDF membranes were incubated with primary antibody overnight at 4 °C and secondary antibody at room temperature for 1 h. Primary antibodies used include ATP5L (Sigma, HPA044629; 1:1,000 dilution), ATP5J (Proteintech, 14114-1-AP; 1:200 dilution), ATP5B (Abcam, ab128743; 1:1,000 dilution), ATP5A (Proteintech, 14676-1-AP; 1:1,000 dilution), OSCP (Bethyl, 50-157-0377; 1:1,000 dilution), c subunit (Abcam, ab180149; 1:1,000 dilution), cyclophilin F (Abcam, ab110324; 1:1,000 dilution), anti-NDUFS1 (Abcam, ab169540; 1:5,000), SDHA (Abcam, ab14715; 1:1,000 dilution), anti-UQCRC1 (Sigma-Aldrich, SAB2702301; 1:1,000), Cox IV (Cell Signaling 11967; 1:1,000 dilution), and GAPDH (Sigma, G8795; 1:5,000 dilution).

### Mitochondria Isolation for BN-PAGE Western Blotting.

#### HAP1 cells.

Two confluent 15 cm plates of HAP1 cells were harvested by scraping and suspended in sucrose buffer for homogenization with a power drill (10×). Cell debris was removed after centrifugation at 600 × g for 10 min at 4 °C. Total lysate was collected and centrifuged at 10,000 × g for 15 min at 4 °C to isolate mitochondria. Then, 30 µg of mitochondria was incubated in an extraction buffer (5% digitonin; 4× BN sample buffer; protease inhibitors; water) for 15 min and then centrifuged at 10,000 × g for 10 min at 4 °C. The supernatant was collected and LB buffer (5% G-250; 4× BN sample buffer; water) was added before loading onto a blue native gel.

#### Isolated cardiac mitochondria.

Hearts were isolated and sliced before homogenization with power drill (40×). Cell debris was removed after centrifugation at 600 × g for 10 min at 4 °C. Total lysate was collected and centrifuged at 8,000 × g for 10 min at 4 °C to isolate mitochondria. Then, 30 µg of mitochondria was incubated in an extraction buffer for 15 min and then centrifuged at 15,000 × g for 5 min at 4 °C. The supernatant was collected, and LB buffer was added before loading onto a blue native gel.

### ATP Assays.

HAP1 cells were seeded overnight in a 10-cm plate. Cells were harvested the next day and transferred in media suspension to a 96-well plate at a concentration of 20,000 cells/well. ATP was measured using an Enzylight adenosine diphosphate (ADP)/ATP Ratio Assay Kit (BioAssay Systems) with a TECAN 1000 luminescent plate reader. Serial dilutions of ATP standard solution (200 nM, 20 nM, 2 nM, 0.2 nM, 0.02 nM) were used to determine absolute molar quantifications of ATP per cell.

### Mitochondrial Membrane Potential.

HAP1 cells were seeded in 12-well plates overnight. Next day, cells were stained with tetramethylrhodamine ethyl ester (ThermoFisher) at a final concentration of 50 nM. Cells were harvested and IMM potential was monitored by flow cytometry (BD LSRII) at excitation and emission wavelengths 530 nm and 575 nm, respectively.

### Seahorse Extracellular Flux Analysis.

HAP1 cells were seeded overnight into Seahorse XF96 V3 PS cell culture microplate (Agilent, 101085-004). The next day, the cells were washed twice with assay medium [DMEM for XF supplemented with 1 mM pyruvate, 2 mM glutamine, 10 mM glucose (Agilent)]. After the wash, 180 µL of the assay media was pipetted into each well and the plate was placed in 37 °C non-CO_2_ incubator for at least 1 h prior to the start of the assay. The Seahorse protocol was set up following standard assay conditions (1.5 min mix, 1.5 min wait, 4 min measure), 4 cycles at baseline, 3 cycles each after oligomycin (1.5 µM), FCCP (1 µM), and rotenone (2 µM)/antimycin A (4 µM) injections. The data was normalized based on total protein concentration of each well determined using Bradford reagent. The normalized data were analyzed using Agilent’s Seahorse XF Cell Mito Stress Test Report Generator.

### Mitochondrial Patch Clamping.

HAP1 cell lines at 80% confluency were harvested and mitochondria were isolated as previously described (21). Mitoplasts were spontaneously formed by diluting isolated mitochondria 100-fold in a patching medium (see below) for 10 min, which caused controlled swelling and rupture of the outer membrane. Mitoplasts could be easily distinguished from intact mitochondria as they appeared as larger and more translucent ring-like structures studded by a curled outer membrane. After the formation of a seal using micropipettes with approximately 0.3-μm tips and resistances of 10 to 30 MΩ at room temperature, inner membrane patches were excised from mitoplasts by bath perfusion with a patching medium (150 mM KCl, 5 mM HEPES, 1.25 mM CaCl_2_, 1 mM EGTA, 5 mM succinate, 2 µM rotenone, pH 7.4). To ascertain mPTP identity, peak currents were routinely monitored before and after addition of freshly diluted cyclosporine A (5 to 20 µM) or the protein import peptide hCoX-IV_(1–22)_. Patches that were sensitive to hCoX-IV_(1–22__)_ were excluded from the analysis. Voltage was clamped with a Dagan 1200 amplifier and reported as pipette potentials. Permeability was typically determined from stable current levels and/or total amplitude histograms of 30 s of data at +20 mV. pClamp version 8 (Axon Instruments) and WinEDR v2.3.3 (Strathclyde Electrophysiological Software) were used for current analysis as previously described (60). The sample rate was 5 kHz with 1 to 2 kHz filtration.

## Supplementary Material

Appendix 01 (PDF)

## Data Availability

All study data are included in the main text and/or *SI Appendix*.
