# Supplementary material for: The mitochondrial ATP synthase is a negative regulator of the mitochondrial permeability transition pore
Source: Proc Natl Acad Sci U S A. 2023 Dec 13;120(51):e2303713120. doi: 10.1073/pnas.2303713120 (PMC10743364; doi:10.1073/pnas.2303713120)
Supplement: Supplementary file 1 — Appendix 01 (PDF) [file pnas.2303713120.sapp.pdf]

PNAS

Supporting Information for

The mitochondrial ATP synthase is a negative regulator of the mitochondrial permeability transition pore

Ryan Pekson[1,3]*, Felix G. Liang[2,3]*, Joshua L. Axelrod[2,3]*, Jaehoon Lee[1,3], Dongze Qin[1,3], Andre J.H. Wittig[2,3], Victor M. Paulino[2,3], Min Zheng[1,3], Pablo M. Peixoto[4], Richard N. Kitsis[1,2,3+]

Departments of Medicine[1], Cell Biology[2], and Wilf Family Cardiovascular Research Institute[3], Albert Einstein College of Medicine, Bronx, NY; Baruch College and Graduate Center, City University of New York, New York, NY[4]

* These authors contribute equally to this work.

[+]Correspondence:

Dr. Richard N. Kitsis

richard.kitsis@einsteinmed.edu

This PDF file includes:

Figures S1 to S4 with legends

Table S1

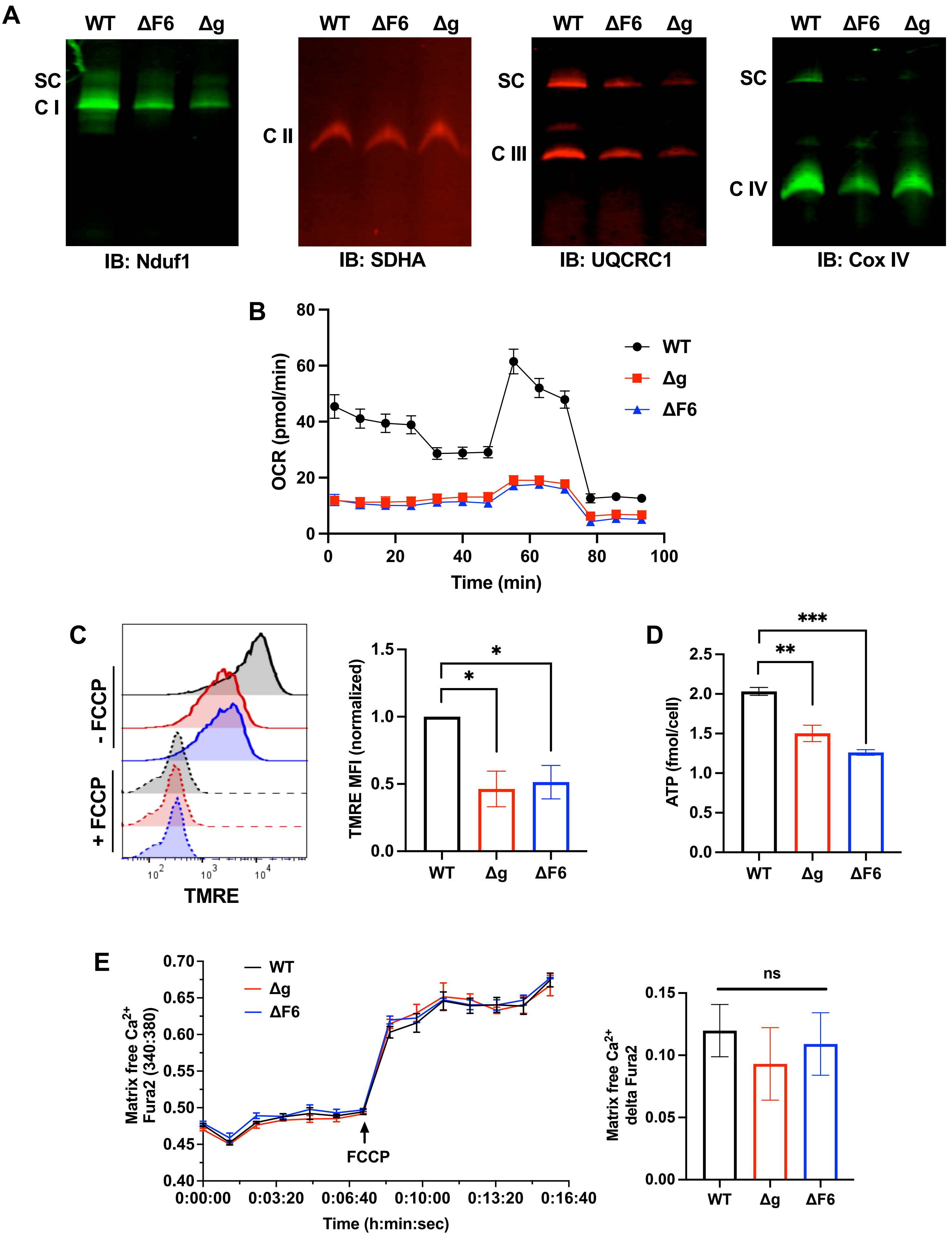
A
WT
ΔF6
Δg
SC
C I
IB: Nduf1
C II
IB: SDHA
C III
IB: UQCRC1
C IV
IB: Cox IV
B
OCR (pmol/min)
Time (min)
C
- FCCP
+ FCCP
TMRE
TMRE MFI (normalized)
D
ATP (fmol/cell)
E
Matrix free Ca2+ Fura2 (340:380)
FCCP
Time (h:min:sec)
Matrix free Ca2+ delta Fura2
ns

Figure S1. Characterization of HAP1-ΔF6 and HAP1-ΔF6 HAP1 cells. (A) Western blot of BN-PAGE assessing Complex I-IV. C I, complex I; C II, complex II; C III, complex III; C IV, complex IV; SC, supercomplex. (B) Cellular oxygen consumption rates (OCR) measured in HAP1-WT, HAP1-Δg and HAP1-ΔF6 cells. Data represent mean ± s.e.m., *n*=4 wells per group. (C) Mitochondrial membrane potential assessed by TMRE in HAP1-WT, HAP1-Δg and HAP1-ΔF6 cells. Data represent mean ± s.e.m., *n*=3 independent experiments. (D) ATP measurements in HAP1-WT, HAP1-Δg and HAP1-ΔF6 cells. Data represent mean ± s.e.m., *n*=3 independent experiments. (E) Measurements of mitochondrial Ca^{2+} content in HAP1-WT, HAP1-Δg and HAP1-ΔF6 cells. Data represent mean ± s.e.m., *n*=4 independent experiments. Statistical analysis were performed using one-way ANOVA. *, $p < 0.05$; **, $p < 0.01$; ***, $p < 0.001$.

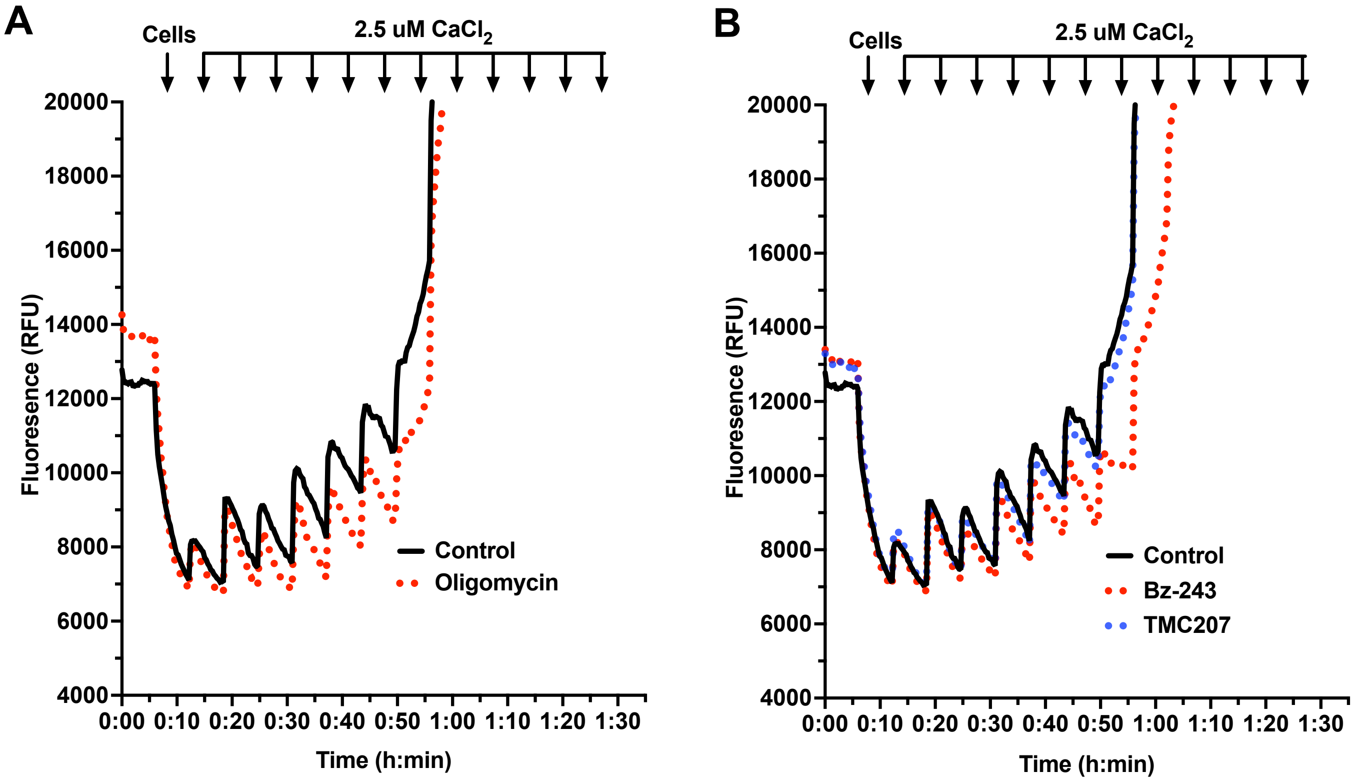

Figure S2. Effect of ATP synthase chemical inhibitors on mPTP opening. Calcium retention capacity (CRC) assay for Ca^{2+} induced mPTP opening in permeabilized HAP1 cells treated with (A) 10μM oligomycin and (B) 20μM Bz-243 or 20μM TMC207.

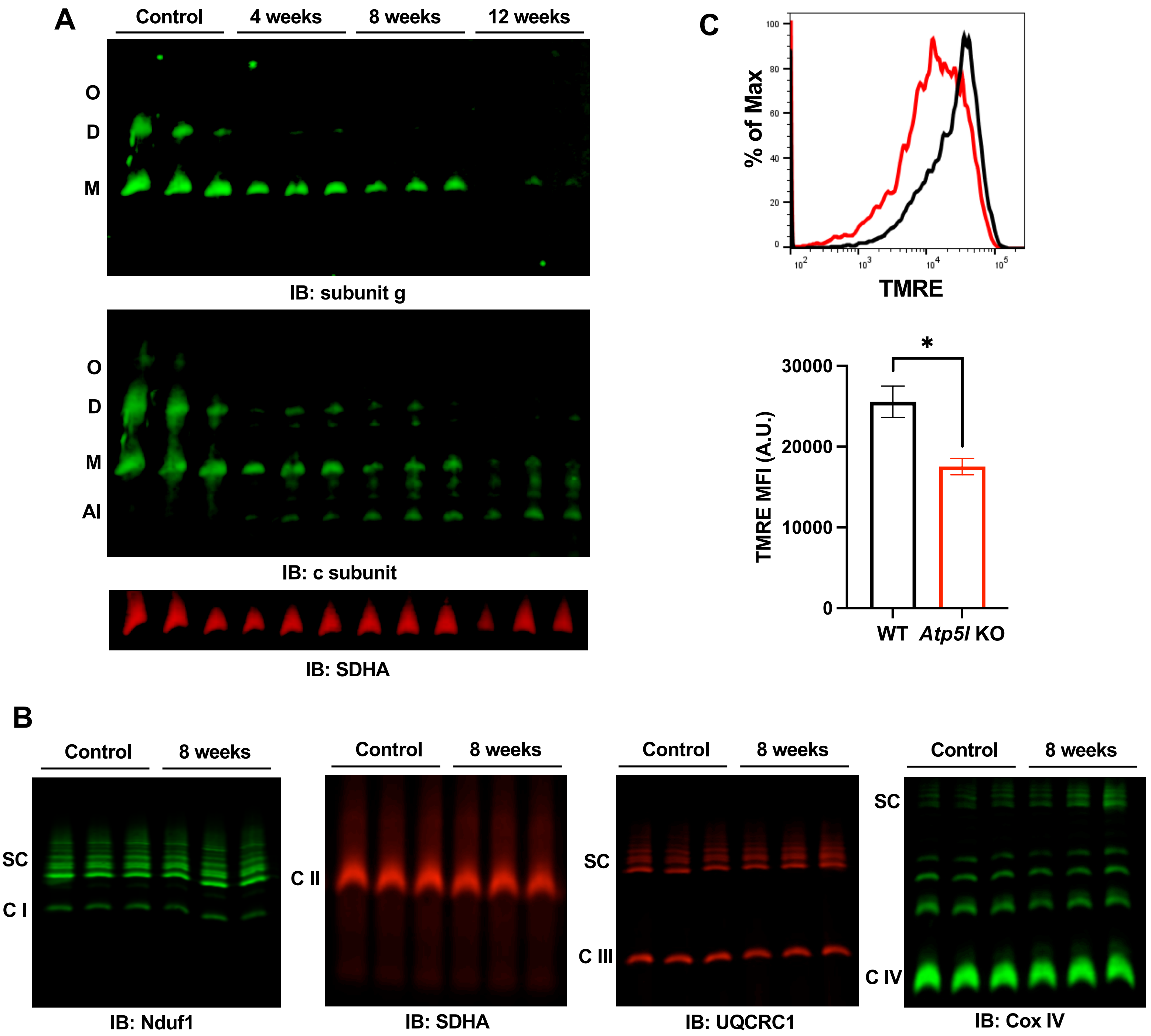

Figure S3. Characterization of *Atp5l* KO mice. (A) Western blot of BN-PAGE for assessing ATP synthase complexes in cardiac mitochondria from WT and *Atp5l* KO mice at the indicated weeks post-TMX. O, oligomers; D, dimers; M, monomers; AI, assembly intermediates. (B) Western blot of BN-PAGE for assessing Complex I-IV. C I, complex I; C II, complex II; C III, complex III; C IV, complex IV; SC, supercomplex. (C) Mitochondrial membrane potential assessed by TMRE in isolated cardiac mitochondria from WT and *Atp5l* KO mice. Data represent mean ± s.e.m., n = 3 WT mice, 3 *Atp5l* KO mice.

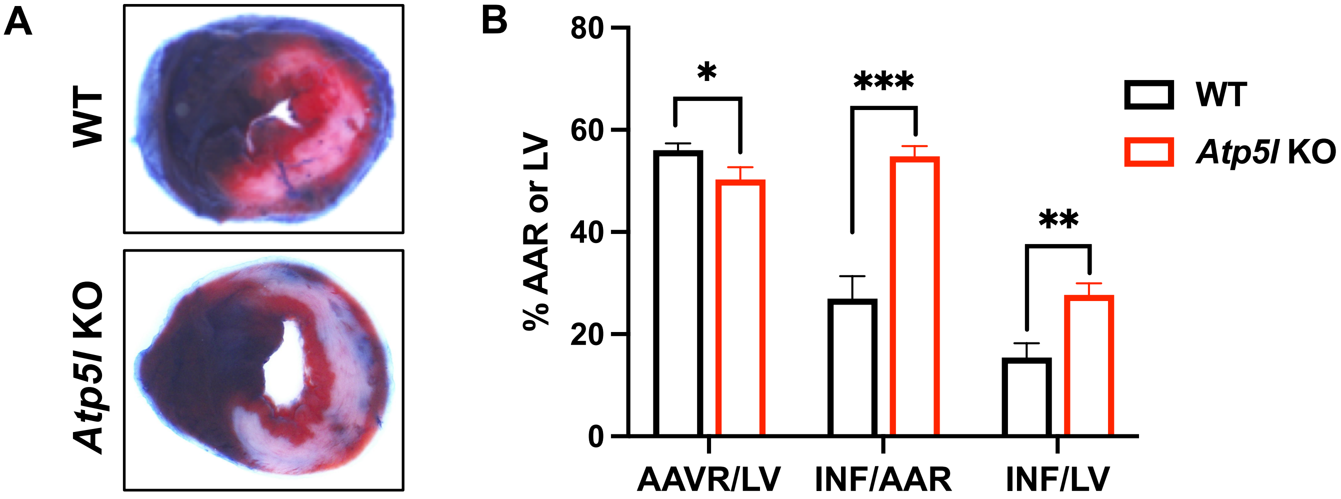

Figure S4. Myocardial I/R in female WT and *Atp5l* KO mice. (A) Representative images of TTC staining to assess infarct size in female WT and *Atp5l* KO mice subjected to myocardial I/R at week 8-10 post-TMX. (B) Quantification of AAVR/LV, INF/AAR, and INF/LV following myocardial I/R in female WT and *Atp5l* KO mice. All data represent mean ± s.e.m., n = 7 female WT mice, 5 female *Atp5l* KO mice. Statistical analysis were performed using two-tailed Student's t test. *, $p < 0.05$; **, $p < 0.01$; ***, $p < 0.001$.

Table S1. Baseline echocardiographic parameters of cardiomyocyte-specific *Atp5l* knockout mice at 8-weeks post-gene deletion

Parameter	Wild type (N=9)	*Atp5l* $^{-/-}$ (N=10)	P value
LVEDV (µl)	55.1±2.2	57.5±2.5	0.07
LVESV (µl)	20.4±1.3	23.1±1.5	0.17
EF (%)	60.3±1.4	59.9±1.4	0.84
IVS(d) (mm)	1.05±0.03	0.98±0.02	0.14
IVS(s) (mm)	1.49±0.04	1.41±0.04	0.22
PW(d) (mm)	0.79±0.03	0.75±0.02	0.33
PW(s) (mm)	1.13±0.02	1.07±0.03	0.17
Heart rate (BPM)	396.3±16.6	431.9±9.8	0.09

LVEDV – left ventricular end diastolic volume; LVESD – left ventricular end systolic volume; EF – ejection fraction; IVS(d) – thickness interventricular septum in diastole; IVS(s) – thickness interventricular septum in systole; PW(d) – thickness posterior wall in diastole; PW(s) – thickness posterior wall in systole. BPM – beats per minute. Data represent mean ± s.e.m. Statistical analyses were performed using two-tailed Student's *t* test.
